# Deoxygedunin, a Natural Product with Potent Neurotrophic Activity in Mice

**DOI:** 10.1371/journal.pone.0011528

**Published:** 2010-07-13

**Authors:** Sung-Wuk Jang, Xia Liu, Chi Bun Chan, Stefan A. France, Iqbal Sayeed, Wenxue Tang, Xi Lin, Ge Xiao, Raul Andero, Qiang Chang, Kerry J. Ressler, Keqiang Ye

**Affiliations:** 1 Department of Pathology and Laboratory Medicine, Emory University School of Medicine, Atlanta, Georgia, United States of America; 2 School of Chemistry and Biochemistry, Georgia Institute of Technology, Atlanta, Georgia, United States of America; 3 Department of Emergency Medicine, Emory University School of Medicine, Atlanta, Georgia, United States of America; 4 Department of Otolaryngology and Cell Biology, Emory University School of Medicine, Atlanta, Georgia, United States of America; 5 Centers for Disease Control and Prevention, Atlanta, Georgia, United States of America; 6 Institute of Neurosciences, Animal Physiology Unit, Autonomous University of Barcelona, Barcelona, Spain; 7 Department of Genetics and Neurology, Waisman Center, University of Wisconsin-Madison, Madison, Wisconsin, United States of America; 8 Psychiatry and Behavioral Sciences, Howard Hughes Medical Institute, Emory University School of Medicine, Atlanta, Georgia, United States of America; Wayne State University School of Medicine, United States of America

## Abstract

Gedunin, a family of natural products from the Indian neem tree, possess a variety of biological activities. Here we report the discovery of deoxygedunin, which activates the mouse TrkB receptor and its downstream signaling cascades. Deoxygedunin is orally available and activates TrkB in mouse brain in a BDNF-independent way. Strikingly, it prevents the degeneration of vestibular ganglion in BDNF −/− pups. Moreover, deoxygedunin robustly protects rat neurons from cell death in a TrkB-dependent manner. Further, administration of deoxygedunin into mice displays potent neuroprotective, anti-depressant and learning enhancement effects, all of which are mediated by the TrkB receptor. Hence, deoxygedunin imitates BDNF's biological activities through activating TrkB, providing a powerful therapeutic tool for treatment of various neurological diseases.

## Introduction

Neurotrophins (NT), a family of secreted proteins, are growth factors that regulate the development and maintenance of the peripheral and the central nervous systems [1]. Brain-derived neurotrophic factor (BDNF) is a member of the neurotrophin family, which includes nerve growth factor (NGF), NT-3 and NT-4/5 [2]. Neurotrophins exert their biological functions on neurons through two transmembrane receptors: the p75 neurotrophin receptor (p75NTR) and the Trk receptor tyrosine kinases (NGF binds to TrkA, BDNF and NT-4/5 bind to TrkB, and NT-3 preferentially binds to TrkC) [3], [4]. Structurally, the extracellular domain of Trk receptors consists of a cysteine-rich cluster (CC1), followed by three leucine-rich repeats, another CC2 and two immunoglobulin (Ig)-like domains, which are involved in ligand binding. The cytoplasmic domain consists of a tyrosine kinase domain surrounded by several tyrosines. BDNF binding to TrkB triggers its dimerization through conformational changes and autophosphorylation of tyrosine residues in its intracellular domain, resulting in activation of the three major signaling pathways involving mitogen-activated protein kinase (MAPK), phosphatidylinositol 3-kinase (PI3K) and phospholipase C-γ (PLC-γ). BDNF protects hippocampal neurons from glutamate toxicity and rescues cerebellar neurons from programmed cell death [5], [6]. BDNF reduces ischemic injury [7], [8] and has been shown to improve functional recovery and postinjury regeneration [9]. Moreover, BDNF is of particular therapeutic interest because of its neurotrophic actions on neuronal populations involved in several neurodegenerative diseases, including peripheral sensory neuropathies [10]; amyotrophic lateral sclerosis (ALS) [11]; Parkinson's disease (PD) and Alzheimer's disease (AD) [12]. The preclinical evidence strongly supports the idea that BDNF might be useful as a therapeutic agent for a variety of neurological disorders. However, the clinical trials with recombinant BDNF are disappointing [13], [14]. Presumably, this is due to the poor pharmacokinetics of BDNF. So far, a few categories of TrkB agonists have been reported, including monoclonal antibodies [15] and peptide mimetics [16], [17]. However, none of these agents have been successfully developed to fully mimic BDNF and to act as potent and selective *in vivo* agonists of TrkB.

In order to identify small molecules that mimic the neurotrophic activities of BDNF, we developed a cell-based apoptotic assay using a cell permeable fluorescent dye MR(DERD)2, which turns red upon caspase-3 cleavage in apoptotic cells. Employing this assay, we have successfully identified TrkA agonist gambogic amide [18]. We then utilized a murine cell line T48, which was derived from basal forebrain SN56 cells that contain undetectable TrkB. T48 cells are TrkB stably transfected SN56 cells. Using this caspase-activated fluorescent dye as a visual assay, we have now screened thousands of compounds. Numerous compounds selectively protected TrkB expressing T48, but not parental SN56 cells lacking TrkB from Staurosporine-initiated apoptosis. This indicates that these compounds might act either directly through TrkB receptor or its downstream signaling effectors. The first round of positive hits was subsequently analyzed on primary hippocampal neurons for TrkB activation and neuronal survival, which was followed by ligand binding and dimerization assays for further characterization. Recently, we have reported that 7,8-dihydroxyflavone (7,8-DHF) acts as a potent TrkB agonist [19].

As described in this manuscript, this screen identified a number of gedunin derivatives. Gedunin, a tetranortriterpenoid isolated from the Indian neem tree (Azadirachta indica), has demonstrated the ability to exhibit antimalarial, insecticidal, and most recently anticancer activity [20], [21], [22]. The antitumor activity of gedunin was explored through the use of the connectivity map [23]. Lamb et al. found, via high connectivity scores with GDA, 17-AAG, and 17-DMAG, that gedunin exhibited its antiproliferative activity through Hsp90 modulation. Gedunin was recently shown to manifest anticancer activity via inhibition of the 90 kDa heat shock protein (Hsp90) folding machinery and to induce the degradation of Hsp90-dependent client proteins similar to other Hsp90 inhibitors. The mechanism of action by which gedunin induces client protein degradation remains undetermined, however, prior studies have demonstrated that it does not bind competitively versus ATP. In this report, we show that deoxygedunin also functions as a TrkB agonist, which binds to the extracellular domain (ECD) of TrkB and stimulates its dimerization and autophosphorylation. Moreover, deoxygedunin selectively activates TrkB but not TrkA or TrkC in BDNF independent fashion, and prevents neuronal apoptosis in a TrkB dependent manner. Strikingly, administration of deoxygedunin prevents vestibular ganglion loss in BDNF −/− pups. Further, deoxygedunin displays robust neuroprotection and antidepressant activities and enhances acquisition of conditioned fear. Thus, deoxygedunin is a TrkB agonist and exerts its neuroprotection through activating TrkB.

## Results

### Identification of gedunin derivatives as survival enhancers

**Figure 1:**
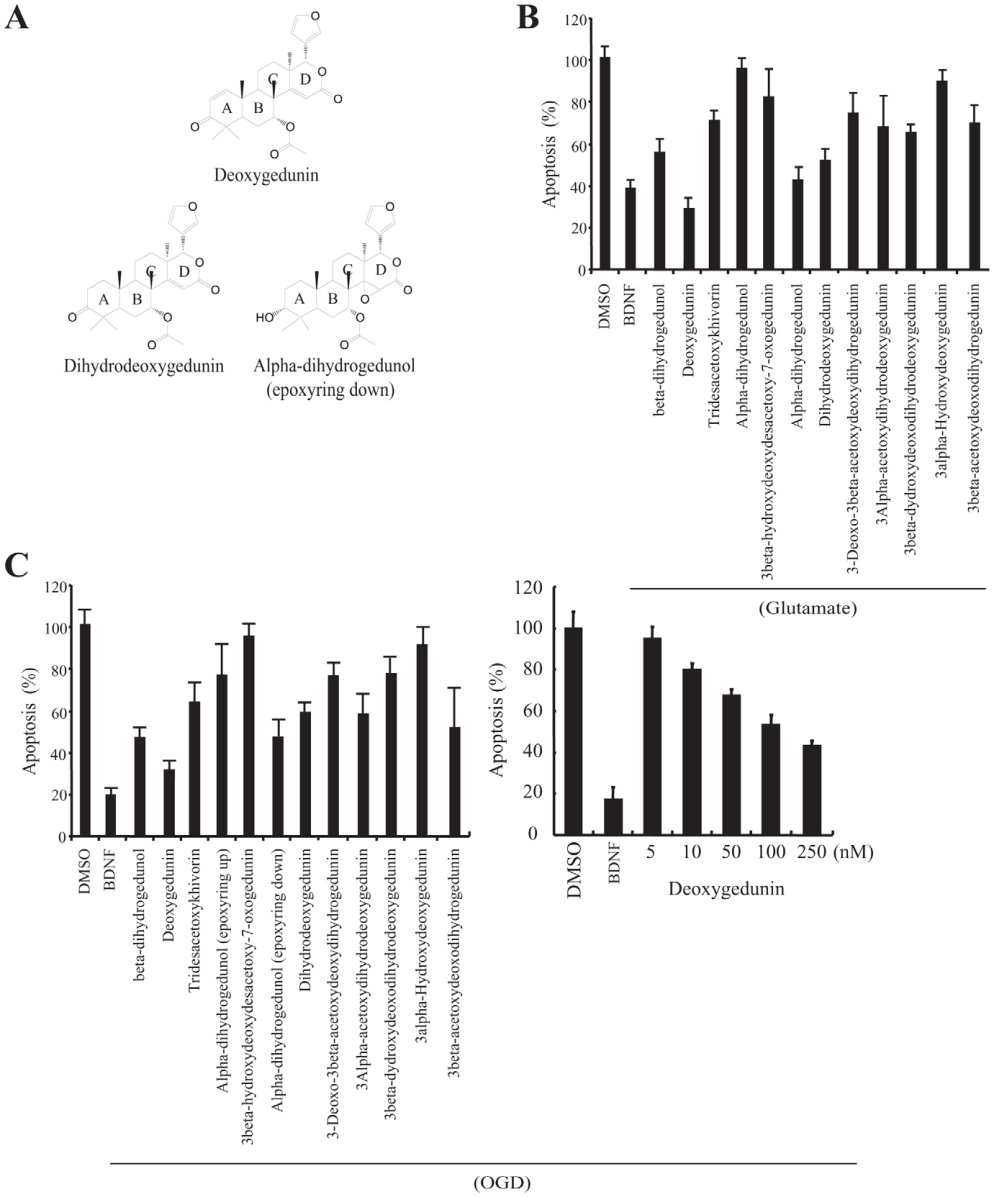
Deoxygedunin protects neurons from apoptosis and it activates TrkB by autophosphorylation. (**A**) Chemical structures of deoxygedunin and two other derivatives. (**B**) Gedunin derivatives suppress glutamate-provoked neuronal cell death. Hippocampal neurons were pretreated with 0.5 µM gedunins for 30 min, followed by 50 µM glutamate treatment for 16 h. Quantitative analysis of neuronal apoptosis was conducted. (**C**) Deoxygedunin inhibits OGD-triggered neuronal apoptosis in a dose-dependent manner.

To search for small molecular TrkB agonists, we developed a cell-based assay using T48 cells, which are TrkB stable transfected SN56 cells, based on the anti-apoptotic action of TrkB signalings. Among 66 positive hits, four are gedunin derivatives. The library also contains numerous gedunin derivatives, which were inactive. The chemical structures of the representative gedunin compounds are depicted in Figure S1. To compare the apoptosis inhibitory activity, we preincubated these compounds (0.5 µM) with hippocampal neurons for 30 min, followed by 50 µM glutamate for 16 h. Among the 12 gedunin derivatives, deoxygedunin displayed the most robust protective effect, followed by alpha-dihydrogedunol (epoxy ring down) and dihydrodeoxygedunin. However, other gedunins barely suppressed apoptosis (Figure 1A & B). The neurons with both cleaved caspase (MR(DEVD)2 red cells) and condensed nuclei (DAPI staining) were counted as apoptotic cells. OGD (Oxygen-Glucose Deprivation) is an *in vitro* model for ischemia stroke. BDNF reduces ischemic injury [7], [8]. To explore whether deoxygedunin exerts any neuroprotective action against OGD, we pretreated hippocampal neurons with 0.5 µM of gedunin derivatives for 30 min, followed by 3 h OGD stimulation (the culture medium was replaced by glucose-free medium, and cells were transferred to a humidified incubation chamber flushed by a gas mixture of 95% N_2_ and 5% CO_2_ at 37°C). Deoxygedunin, alpha-dihydrogedunol (epoxy ring down) and dihydrodeoxygedunin exhibited potent protective effects on hippocampal neurons under OGD. Titration assay reveals that deoxygedunin protected neurons in a dose-dependent manner (Figure 1C).

### Deoxygedunin activates TrkB and protects neurons from apoptosis

**Figure 2:**
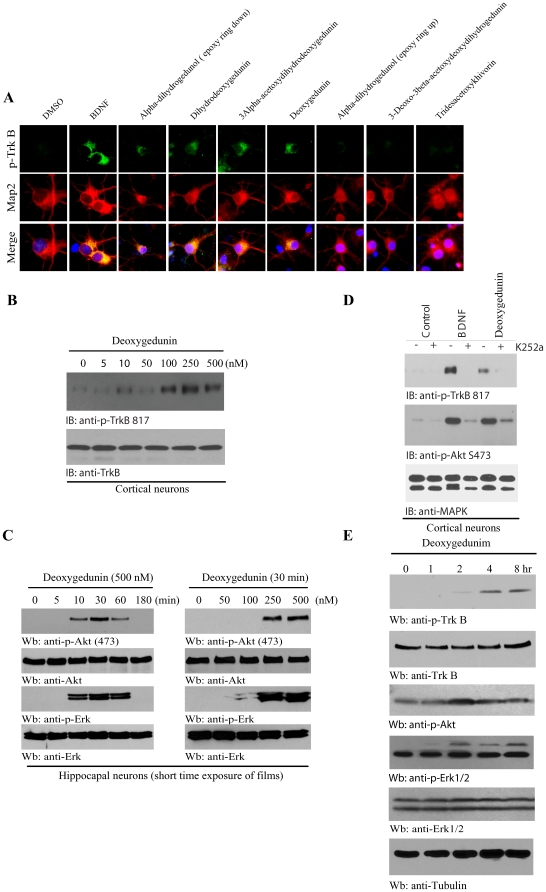
Deoxygedunin activates TrkB and protects neurons from apoptosis. (**A**) Deoxygedunin activates TrkB in primary hippocampal neurons. Hippocampal neurons were treated with 500 nM gedunin derivatives for 30 min and neurons were fixed and immunostained with rabbit polyclonal anti-p-TrkB (816) (1∶100) and anti-MAP2. The nuclei were stained with DAPI. BDNF and a few gedunin derivatives selectively triggered TrkB phosphorylation in neurons. (**B**) Deoxygedunin triggers TrkB activation in primary neurons. Rat cortical neurons were treated with various concentrations of deoxygedunin for 30 min. Neuronal lysates were subjected to immunoblotting analysis by mouse monoclonal anti-p-TrkB (817)(1∶20,000). Equal amount of TrkB was loaded (anti-TrkB from Biovision, 1∶1,000) (lower panel). (**C**) Deoxygedunin provokes Akt and Erk1/2 activation in primary neurons in a dose and time-dependent manner. Mouse monoclonal anti-TrkB 817 was used at 1∶20,000 dilution. (**D**) K252a blocks deoxygedunin's agonistic effect on TrkB. Cortical neurons were pretreated with K252a (100 nM) for 30 min, followed by BDNF (100 ng/ml) or deoxygedunin (500 nM) for 30 min. Cell lysates were analyzed by immunoblotting. (**E**) Deoxygedunin activates TrkB in mouse brain. Deoxygedunin (5 mg/kg) was intraperitoneally injected into mice and mouse brains were dislocated at different time points. Brain lysates were analyzed by immunoblotting.

To examine whether deoxygedunin triggers TrkB signaling cascade, we conducted immunofluorescent staining and immunoblotting assays. The positive gedunin derivatives elicited TrkB activation in rat hippocampal neurons (Figure 2A). Among a few gedunin positive hits, deoxygedunin elicited the strongest stimulatory effect. Hence, we selected it and focused on this compound. Deoxygedunin activated TrkB in primary neuronal cultures in a dose-dependent manner (Figure 2B). In hippocampal neurons, deoxygedunin prominently provoked both Erk1/2 and Akt activation with a time course (Figure 2C, left panels). It stimulated both Erk1/2 and Akt activation in a dose-dependent manner. The minimal required drug concentration was about 100-250 nM (Figure 2C, right panels). Hence, the activation patterns of TrkB receptor and its downstream effectors including Akt and Erk1/2 by deoxygedunin were tightly correlated. K252a is a Trk receptors inhibitor. Pretreatment of K252a substantially blocked deoxygedunin-triggered TrkB activation in cortical neurons (Figure 2D), indicating that deoxygedunin can provoke TrkB autophosphorylation. Deoxygedunin-provoked downstream Akt signalings were also reduced by K252a. To assess whether deoxygedunin can provoke TrkB activation in the brain, we injected mice (i.p.) with a dose of 5 mg/kg for various time points. TrkB was selectively phosphorylated in the brain 2 h after injection, and peaked at 4–8 h, so was the downstream effectors Akt and Erk1/2 activation (Figure 2E, left panels), suggesting that deoxygedunin can penetrate the brain-blood barrier and stimulate TrkB activation. Deoxygedunin was also orally bioactive in provoking TrkB activation (Figure S2A). RT-PCR analysis revealed no change of TrkA or TrkB in mouse brain upon deoxygedunin treatment (Figure S2B), indicating that deoxygedunin stimulates TrkB activation independent of Trk receptor transcriptional alteration. Mouse monoclonal P-TrkB 817 antibody recognized both human and mouse TrkB phosphorylation in addition to rat TrkB (Figure S2C). Hence, deoxygedunin can strongly trigger TrkB activation *in vitro* and *in vivo*.

### Deoxygedunin binds TrkB ECD and provokes its dimerization

**Figure 3:**
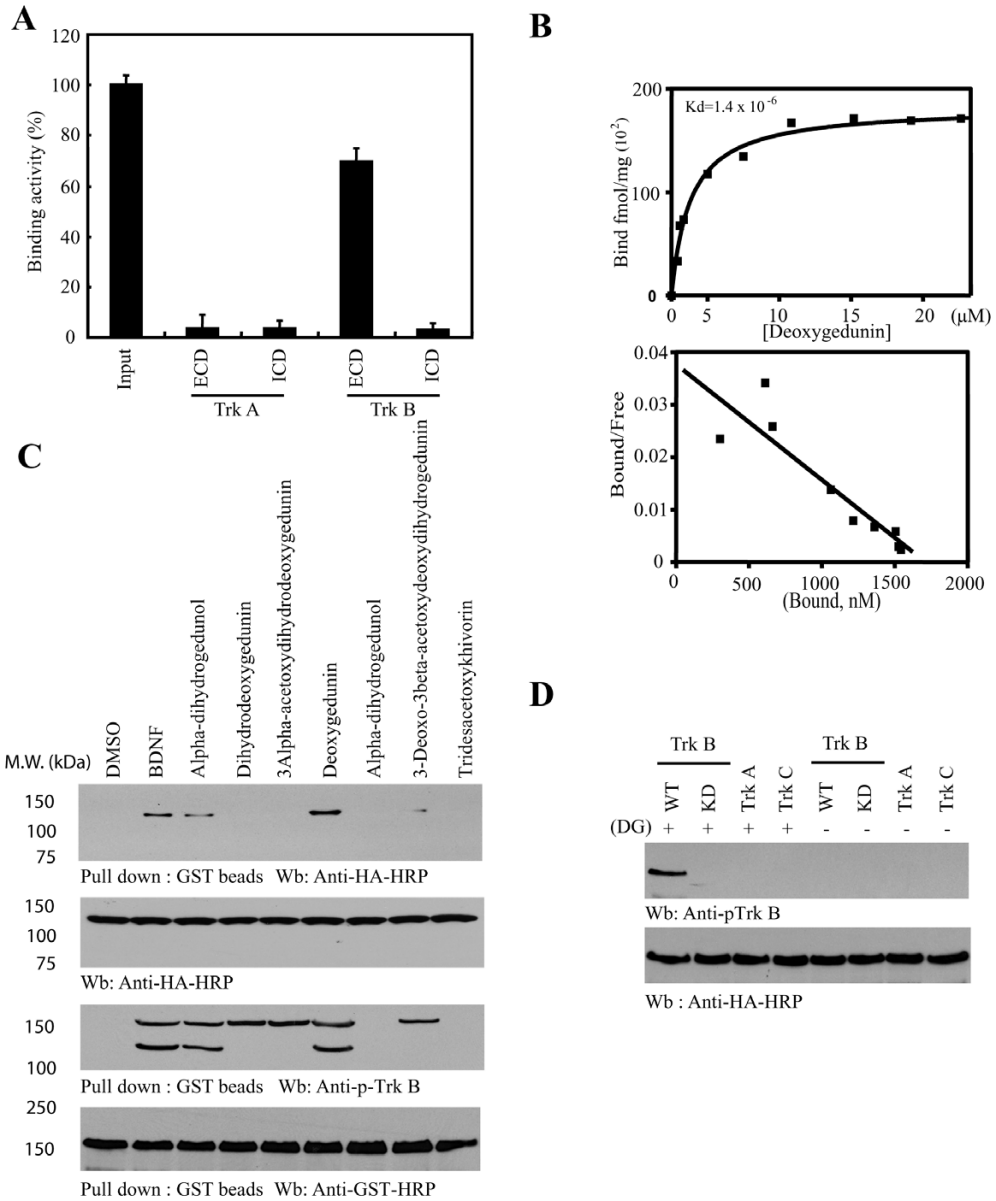
Deoxygedunin binds TrkB ECD and elicits its dimerization. (**A**) [^3^H]deoxygedunin binds the ECD but not ICD of TrkB receptor. *In vitro* binding assay with purified TrkB ECD or ICD (10 µg) and [^3^H]deoxygedunin (upper panel). *In vitro* binding assay with purified fragments of TrkB ECD (10 µg) and [^3^H]deoxygedunin. (**B**) Determination of binding constant. Binding curve (upper panel). Scatchard plot analysis revealed that deoxygedunin binds TrkB with binding constant of 1.4 µM (lower panel). (**C**) Deoxygedunin provokes TrkB dimerization. mGST-TrkB and HA-TrkB were cotransfected into HEK293 cells, treated with 0.5 µM various gedunins for 30 min. GST-TrkB was pulled down by glutathione beads, the coprecipitated proteins were analyzed with anti-HA HRP antibody. (**D**) Deoxygedunin induces TrkB autophosphorylation. HEK 293 cells were transfected with various Trk receptors, followed by deoxygedunin treatment for 30 min. Cell lysates were analyzed by immunoblotting assay.

To determine whether deoxygedunin directly binds TrkB, we conducted a ligand binding assay with [^3^H]deoxygedunin. Filter assay demonstrated that increasing concentrations of [^3^H]deoxygedunin progressively bound TrkB ECD but not ICD, indicating that TrkB ECD but not ICD selectively binds deoxygedunin. In contrast, it did not bind to TrkA at all, indicating it specifically associates with the extracellular domain of TrkB receptor (Figure 3A). Scatchard plot analysis revealed that the ratio of ligand to the receptor is 1∶1 with binding constant K_d_  = 1.4 µM (Figure 3B). To test whether deoxygedunin triggers TrkB dimerization, we cotransfected GST-TrkB with HA-TrkB plasmid into HEK293 cells, and treated the cells with 0.5 µM of various gedunin derivatives for 30 min. GST pull-down assay revealed that deoxygedunin robustly provoked TrkB dimerization with an effect even stronger than BDNF. Moreover, alpha-dihydrogedunol (epoxy ring down) also notably promoted TrkB dimerization (Figure 3C, top panel), fitting with its stimulatory activity on TrkB (Figure 2). The coprecipitated HA-TrkB was also prominently tyrosine phosphorylated (3^rd^ panel). Hence, deoxygedunin directly binds TrkB ECD and triggers its association. Deoxygedunin also elicited tyrosine phosphorylation in TrkB but not in TrkA or TrkC receptor in transfected HEK293 cells. TrkB-KD displayed negligible phosphorylation compared to wild-type TrkB (Figure 3D), indicating that TrkB phosphorylation by deoxygedunin is through the receptor autophosphorylation but not by any other tyrosine kinases. Therefore, deoxygedunin binds to the ECD of TrkB and promotes its association and activation.

### Deoxygedunin protects neurons from apoptosis in a TrkB-dependent manner

**Figure 4:**
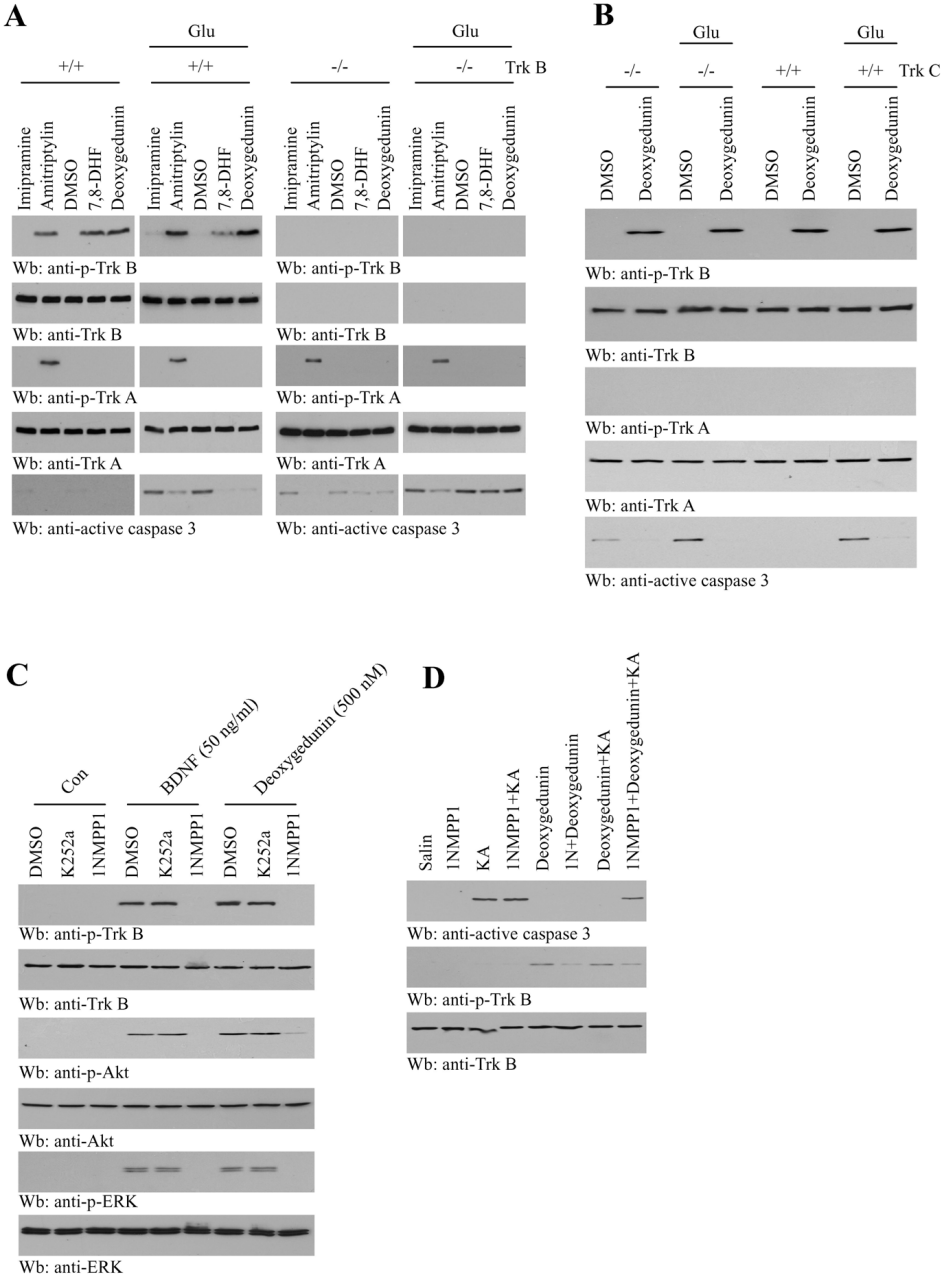
Deoxygedunin protects neurons from apoptosis in a TrkB-dependent manner. (**A**) Deoxygedunin prevents glutamate-triggered neuronal apoptosis in wild-type but not TrkB null neurons. Cortical neurons were prepared from the P0 pups (TrkB +/− x TrkB +/−). The neurons were pretreated with a variety of compounds as indicated for 30 min, followed by 50 µM glutamate for 16 h. The cell lysates were analyzed by immunoblotting with anti-p-TrkB, anti-p-TrkA and anti-active caspase-3 etc. (**B**) Deoxygedunin diminishes caspase-3 activation regardless of TrkC genotype. (**C**) Deoxygedunin selectively activates TrkB F616A, which can be blocked by 1NMPP1. Primary cortical neurons were prepared from TrkB F616A knockin mice. The primary cultures were pretreated for 30 min with either K252a Trk tyrosine kinase inhibitor (100 nM) or 1NMPP1 inhibitor (100 nM), followed by 0.5 µM Deoxygedunin for 30 min. Immunoblotting with various antibodies were performed. (**D**) Deoxygedunin suppresses KA-induced neuronal cell death in TrkB F616A mutant mice, which can be blocked by 1NMPP1. TrkB F616A knockin mice were treated with following reagents: saline, 1NMPP1, kainic acid, 1NMPP1 + kainic acid, deoxygedunin, 1NMPP1 + deoxygedunin, 1NMPP1 + deoxygedunin + kainic acid, as described in experimental section. Immunoblotting was conducted with indicated antibodies.

To determine if deoxygedunin's neuronal protective effect is mediated through TrkB receptor, we prepared cortical neurons from pups of TrkB +/− mice, which were mated to the same genotype. Deoxygedunin specifically activated TrkB but not TrkA receptor in wild-type but not TrkB −/− neurons. 7,8-dihydroxyflavone (7,8-DHF), another positive compound from the screening, also selectively activated TrkB but not TrkA. The tricyclic antidepressant drugs amitriptyline but not imipramine activated both TrkA and TrkB (Figure 4A, top and 3^rd^ panels). Glutamate-provoked caspase-3 activation was substantially blocked by 7,8-DHF and deoxygedunin in wild-type but not TrkB −/− neurons. However, the control compound imipramine failed to blocked caspase-3 activation by glutamate. In contrast, amitriptyline weakly suppressed caspase-3 activation in both wild-type and TrkB −/− neurons (Figure 4A, bottom panels). Thus, deoxygedunin selectively suppressed apoptosis triggered by glutamate in a TrkB-dependent manner. Moreover, deoxygedunin strongly provoked TrkB but not TrkA activation in both wild-type and TrkC knockout neurons (Figure 4B, top panel). Noticeably, the spontaneous caspase-3 activation in TrkC −/− neurons was suppressed by deoxygedunin. Further, glutamate-triggered caspase-3 activation was significantly diminished by deoxygedunin (Figure 4B, bottom panel), demonstrating that it represses neuronal apoptosis is TrkB- but not TrkC-dependent.

To investigate whether the neuroprotective effects of deoxygedunin is reliant on TrkB activation *in vivo*, we employed TrkB F616A knock-in mice, where it has been shown that TrkB F616A can be selectively blocked by 1NMPP1, a TrkB F616A inhibitor, resulting in an effective TrkB-null phenotypes [24]. To assess whether deoxygedunin can mimic BDNF, we prepared cortical neurons from TrkB F616A knock-in mice. Fitting with the previous report, BDNF-provoked TrkBF616A phosphorylation was selectively blocked by 1NMPP1 but not K252a. We made the same finding with deoxygedunin (Figure 4C, top panel). 1NMPP1, but not K252a, blocked BDNF-triggered Akt and Erk1/2 activation. Similarly, 1NMPP1 completely diminished Akt and Erk1/2 activation by deoxygedunin (Figure 4C, 3^rd^ and 5^th^ panels). Since 1NMPP1 selectively inhibits TrkB F616A activation by deoxygedunin, we hypothesized that blockade of TrkB F616A signaling by 1NMPP1 in mice would make the neurons vulnerable to KA-provoked neuronal cell death. We pretreated the mice with 1NMPP1, followed by deoxygedunin and KA treatment. In 5 days, we monitored neuronal cell death in hippocampal region by immunoblottings. As predicted, 1NMPP1, deoxygedunin alone or 1NMPP1 + deoxygedunin combined treatment had no effect on apoptosis in TrkB F616A mice. KA caused marked caspase-3 activation, and pretreatment of 1NMPP1 mildly elevated KA-provoked apoptosis in TrkB F616A, supporting that TrkB signaling is critical for neuronal survival. Deoxygedunin evidently suppressed KA-provoked apoptosis, whereas 1NMPP1 pretreatment greatly diminished deoxygedunin's protective effect in F616A mice. TrkB activation status inversely correlated with TrkB activation by deoxygedunin (Figure 4D, top and middle panels). Hence, these data support that deoxygedunin selectively activates TrkB receptor and enhances neuronal survival in mice in TrkB dependent manner.

### Deoxygedunin activates TrkB in BDNF independent manner and prevents vestibular ganglion loss

**Figure 5:**
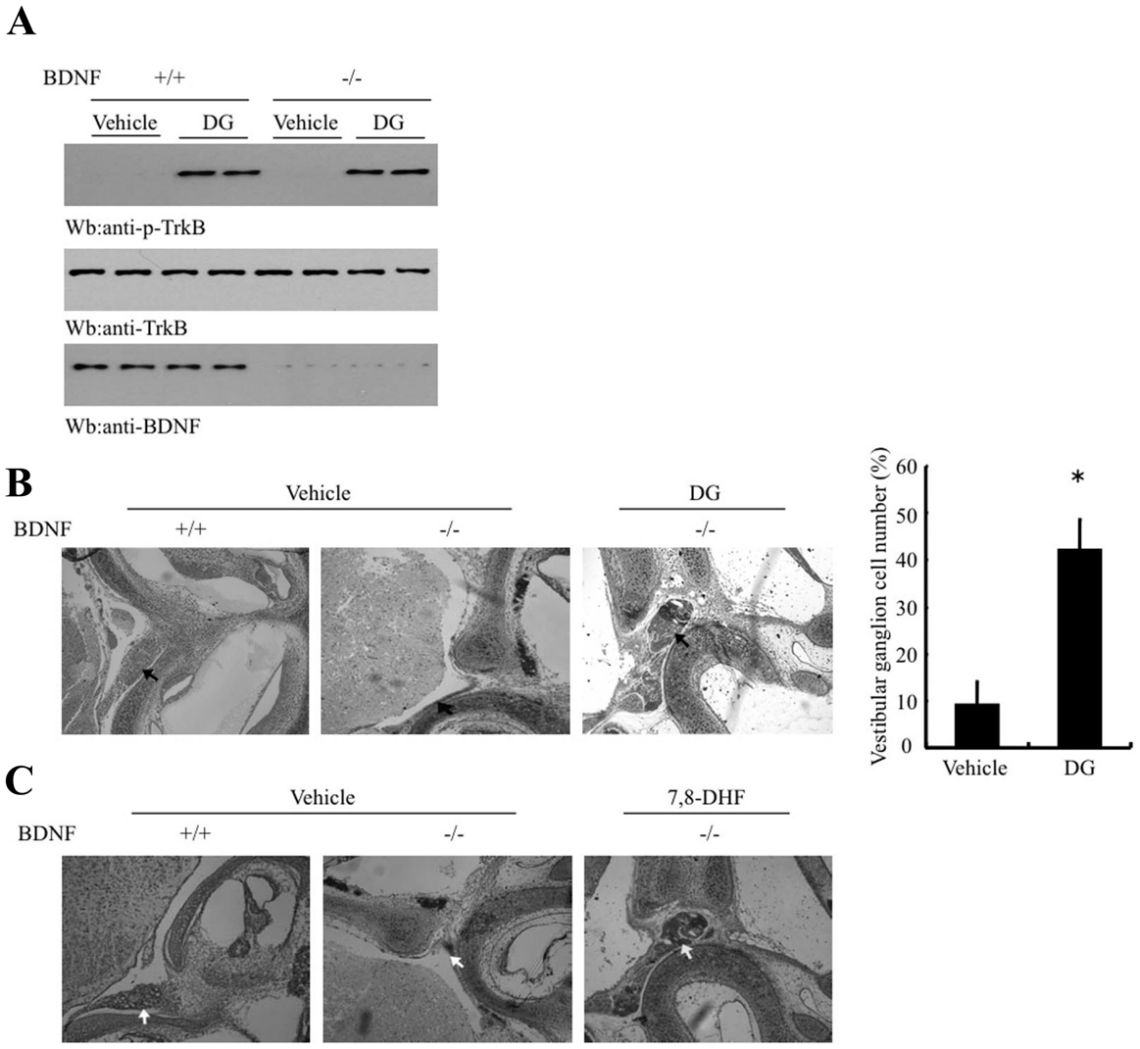
Deoxygedunin activates TrkB in a BDNF-independent manner and prevents vestibular ganglion loss. (**A**) Deoxygedunin triggers TrkB activation in BDNF conditional knockout cortex. 2–3 months old BDNF cortex conditional knockout mice were intraperitoneally injected with 5 mg/kg deoxygedunin. In 4 h, the mice were sacrificed and brain lysates were analyzed by immunoblotting. (**B & C**) Deoxygedunin and 7,8-DHF prevent vestibular ganglion loss in BDNF −/− pups. BDNF +/− mice were mated with the same genotypes of mice. At E7.5 days, the pregnant mothers were administrated with 5 mg/kg deoxygedunin or 7,8-DHF until birth. The neonatal pups continued on drug treatment for 1 or 2 days till death. The inner ear sections were stained with toluidine blue.

To examine whether deoxygedunin activating TrkB involves endogenous BDNF, we employed BDNF conditional knockout mice with BDNF gene deletion limited to cortex, thus allowing normal development. We intraperitoneally injected deoxygedunin (5 mg/kg) into BDNF cortex conditional knockout mice and sacrificed the mice at 4 h. Immunoblotting analysis with the cortical lysates demonstrated robust TrkB activation in both wild-type and BDNF −/− mice (Figure 5A), underscoring that deoxygedunin activates TrkB independent of BDNF. Mutant mice lacking BDNF have severe deficiencies in coordination and balance, associated with excessive degeneration in several sensory ganglia including the vestibular ganglion [25]. To determine whether deoxygedunin rescues the loss of vestibular ganglions in BDNF −/− pups, we bred the conventional BDNF +/− mice with the same genotype mice, and administered deoxygedunin (5 mg/kg, i.p.) to the pregnant mice at day E7.5 until birth. The neonatal pups continued receiving the same dose of deoxygedunin, but BDNF −/− pups continued dying at P1 or P2. Staining of inner ear sections showed that vestibular ganglia were completely lost in most of control vehicle-treated BDNF −/− pups. In contrast, many of deoxygedunin-treated BDNF mutant mice displayed intact vestibular ganglia, similar to the wild-type pups (Figure 5B, left panels). Quantitative analysis demonstrated that 9.1% of vestibular ganglia were detected in vehicle-treated BDNF −/− pups, whereas deoxygedunin treatment increased to 42.2% (Figure 5B, right panel). We made a similar observation with 7,8-DHF (Figure 5C). Therefore, deoxygedunin mimics BDNF and significantly protects vestibular ganglia from degeneration in BDNF -/- pups.

### TrkB agonists have potent antidepressant effect

**Figure 6:**
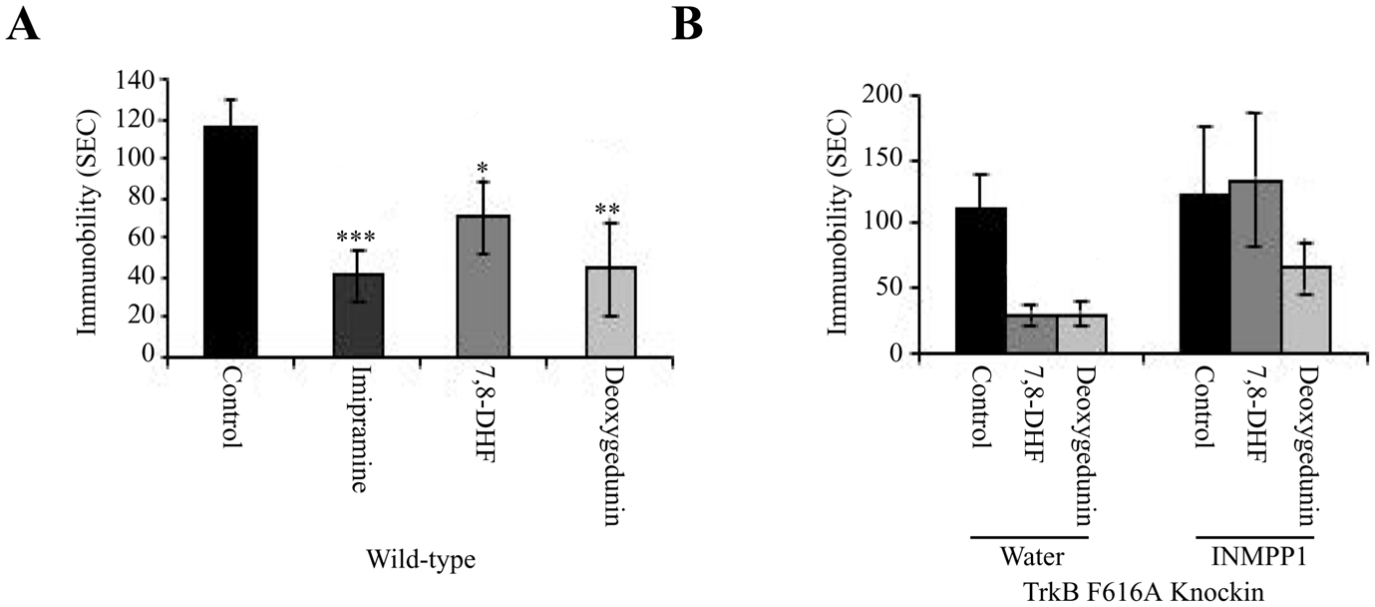
TrkB agonists are robust antidepressants. (**A**) Forced swim test. 2–3-month-old Male mice (n = 8 mice/group) were treated with imipramine (20 mg/kg), deoxygedunin (5 mg/kg), 7,8-DHF (5 mg/kg) and vehicle solvent saline by intraperitoneal injection for 5 days, and subjected to a forced swim test (6 min, immobility recorded in the last 4 min). (**B**) Forced swim test with TrkB F616A knockin mice. Male TrkB knockin mice were given the regular drinking water or 1NMPP1 containing drinking water one day before the drugs. Deoxygedunin/1NMPP1 group is significantly different from the water group but not from control in 1NMPP1. Data are presented as mean ± SEM of n = 8–10 mice/group (**P<0.01 and ***P<0.001 against vehicle control group, One-way ANOVA, Dunnett's test).

BDNF plays an essential role in mediating antidepressants' therapeutic effects [26], [27], [28], [29]. Infusion of exogenous BDNF into hippocampus or brain stem has anti-depressant-like behavioral effects [30], [31]. A forced swim test is broadly used for screening of potential antidepressant drugs and is widely used to measure antidepressant activity [32], [33]. To investigate whether these TrkB agonists mimic BDNF in suppressing depression-like symptoms, we conducted a forced swim test after subchronic treatment of the mice for 5 days with various drugs. When mice were treated with imipramine (20 mg/kg), a tricyclic antidepressant drug, the swimming immobility was significantly decreased. Interestingly, both 7,8-DHF and deoxygedunin (5 mg/kg) reduced the immobility with deoxygedunin revealing a more potent effect (Figure 6A). To assess whether the behavior responses by 7,8-DHF and deoxygedunin are mediated by TrkB receptor, we utilized TrkB F616A knockin mice. The transgenic mice were subjected saline or 1NMPP1 treatment, respectively. No significant difference was observed in the immobility time between saline and 1NMPP1-treated mice. In saline group, 7,8-DHF and deoxygedunin substantially reduced the immobility time; in contrast, 7,8-DHF and deoxygedunin had no significant effect in mice, when TrkB was blocked by 1NMPP1 (Figure 6B), suggesting that inhibition of the TrkB signaling cascade inhibits the antidepressant effect of these drugs. Thus, these data demonstrate that the TrkB agonists mimic BDNF and act as potent antidepressant drugs in mice through activating the TrkB receptor.

### Deoxygedunin enhances acquisition of conditioned fear, a BDNF-dependent learning process

**Figure 7:**
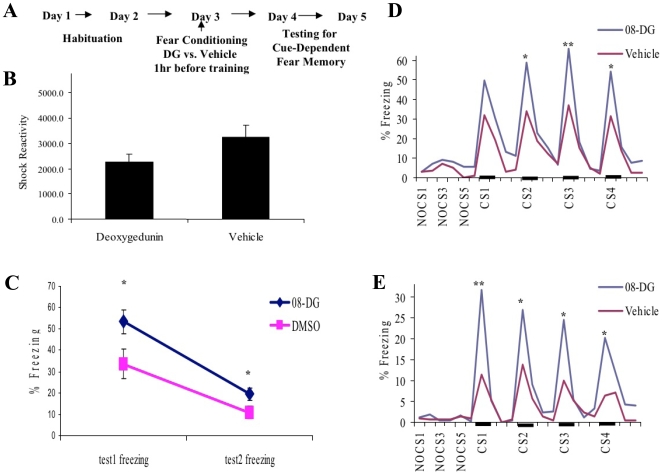
Deoxygedunin enhances acquisition of conditioned fear, a BDNF-dependent learning process. (**A**) Outline of Deoxygedunin (DG) fear conditioning experiment. Mice were handled and habituated to testing context on the first two days, followed by systemic DG (5 mg/kg, i.p.) 1 hr prior to fear conditioning (5, 0.5mA shock-tone pairings), followed by testing in the absence of drug on days 4–5. (**B**) Shock reactivity during fear conditioning, demonstrating that the acute effects of the drug did not affect pain or fear responsiveness. (**C**) Total freezing to the conditioned cue was significantly greater on both the first and second testing day in the mice that received DG with fear conditioning. (**D & E**), Freezing activity during the habituation period and first 4 CS presentations on test day 1 (**D**) or 2 (**E**). There was no difference in animal activity in the test chamber prior to the onset of the conditioned cue, or between cue presentations. However there was significantly increased fear, as measured with conditioned freezing, during conditioned stimuli (CS). Dark bars represent tone conditioned stimulus presentations; **, p<0.01, *, p<0.05 between DG and vehicle groups.

We next wished to examine whether this TrkB agonist would enhance learning in a whole animal model of learning and memory, in which BDNF-dependent TrkB activation is required. Activation of TrkB by BDNF has been repeatedly shown to be associated with, and required for, the acquisition of classical conditioned fear in rodent models [34], [35], [36], [37], [38], [39]. Following habituation to the testing context, 28 adult wild-type, C57BL/6J mice were given systemic injections of deoxygedunin (N = 14, 5 mg/kg, i.p.) or vehicle (N = 14) 1 hr prior to tone-shock fear conditioning in a novel context (Figure 7A). There was no difference between treatment groups in shock reactivity during the fear acquisition training, suggesting that there were no acute effects on pain sensitivity that would affect fear acquisition or later fear expression (p>.1; Figure 7B). Mice were then tested, with no additional drug treatment, for cue-conditioned fear in the previously habituated context on the two days following fear acquisition. The average level of tone-dependent conditioned freezing was significantly different on both testing days (Figure 7C; repeated measures ANOVA, F(1,26) = 6.6, p = .016) suggesting that mice that received deoxygedunin at the time of training had enhanced acquisition or consolidation of the fear memory. To further explore these effects, we examined individual animals' freezing levels throughout the tone-fear testing sessions. We found on both testing day 1 (Figure 7D) and day 2 (Figure 7E) that the enhancement in freezing only corresponded with the periods of tone cue presentation. The mice demonstrated similar levels of locomotor exploratory activity prior to and in-between tone exposure in this context, but the animals that received deoxygedunin during the previous tone-shock fear conditioning demonstrated significantly increased freezing during cued fear presentations (Day 1, repeated measures ANOVA of first 4 CS trials, F(1,26) = 8.1, p<.01; Day 2, F(1,26) = 7.5, p<.01). This increase in fear learning led to a 2–3 fold increase in the level of freezing during the first set of conditioned stimulus (CS) trials examined each day. Together, these results suggest that although deoxygedunin does not affect apparent level of pain or shock reactivity during training, nor does it affect general locomotor activity in the testing situation on subsequent days, the learning event that occurred during training in the presence of systemic deoxygedunin compared with vehicle was acquired or consolidated in a more effective manner. Since cue-dependent fear conditioning is known to require, and be exquisitely sensitive to, BDNF activation of TrkB, these data are consistent with deoxygedunin acting on the TrkB system *in vivo* to enhance cue-dependent fear learning.

## Discussion

In the present study, we have demonstrated that deoxygedunin directly binds the ECD of TrkB and promotes its dimerization and activation. Deoxygedunin provokes TrkB activation in primary neurons and in mouse brain. It strongly protects neurons from apoptosis in a TrkB- dependent manner. Further, it activates TrkB in BDNF conditional knockout mice, indicating that BDNF is not implicated in the stimulatory effect of deoxygedunin. When it is injected in animals, deoxygedunin mimics BDNF and exerts neuroprotective and antidepressant actions and enhances learning processes. Moreover, administration of 7,8-dihydroxyflavone and deoxygedunin into pregnant BDNF +/− mothers substantially rescues vestibular ganglia, which are significantly degenerated in BDNF −/− pups.

To determine which chemical group or moiety is critical for the neurotrophic activity of gedunin, we have conducted a structure-activity relationship study. The results suggest that the epoxy ring confirmation in the D ring is essential for gedunin's agonistic activity. It has to be in a down conformation for the agonist to be effective. Nevertheless, the alpha-beta unsaturated ketone in ring A is variable. Reduction of the unsaturated C = C bond did not impair its stimulatory activity, suggesting that this site can tolerate a potential chemical modification, which might be useful for improving its water solubility and biological activity. When the epoxy group is in down conformation, changing ketone into alcohol did not cripple its agonistic effect either regardless of alpha or beta conformation of the resulting hydroxy group. Nevertheless, deoxygedunin displays the strongest agonistic effect, suggesting that ketone might be more active than alcohol in ring A (Figure S1). We have recently shown that 7,8-DHF and NAS (N-acetylserotonin) potently activate TrkB receptor. Structurally, these two compounds share the hydroxyphenol group. Our structure-activity study in 7,8-DHF shows that the 7-position hydroxyl group is critical for its agonist effect. This position parallels the 5-position hydroxyl group on the indole ring in NAS. Conceivably, these two compounds share the same binding pocket on TrkB ECD. However, they are quite different from deoxygedunin in structure. The latter belongs to terpenoid family members. Deoxygedunin might bind to different motif on TrkB ECD from that of 7,8-DHF and NAS.

Gedunin, a tetranortriterpenoid isolated from the Indian neem tree (Azadirachta indica), and was recently shown to manifest anticancer activity via inhibition of the 90 kDa heat shock protein (Hsp90) folding machinery and to induce the degradation of Hsp90-dependent client proteins similar to other Hsp90 inhibitors [20], [21], [22], [23], [40]. Intraperitoneal injection of deoxygedunin (5 mg/kg) into mice triggers TrkB activation in mouse brain after 2 h and peaked at 4–8 h, indicating that this compound or its metabolites can pass brain blood barrier and has a fairly long effective duration. To monitor its tissue distribution and kinetics of degradation after i.p. injection, we collected different brain regions and body organs 4 h after ^3^H-deoxygedunin administration. ^3^H-labeled compounds were concentrated in olfactory bulb and hippocampus in the brain. Clearly, the brain as a whole organ was the major tissue in the body where ^3^H-labeled compound was accumulated, followed by the lung (Figure S4). Obviously, substantial amount of ^3^H-deoxygedunin and its metabolites remained in the brain 4 h after drug administration, indicating this compound might possess a favorable half-life in the body. Further, it can activate TrkB in mouse brain via oral administration. Nevertheless, deoxygedunin and other derivatives have poor water solubility. In order to alleviate this issue, we are now synthesizing various novel derivatives directly from deoxygedunin that possess better water solubility while maintaining its biological efficacy. Our rationale for modifying the skeleton of deoxygedunin will occur in two phases. In the first phase, we will modify the furan ring in the hopes of increasing solubility. In the second phase, we will modify the ABCD rings of the solubilized deoxygedunin derivatives (from phase one) in order to improve their biological activity.

Deoxygedunin binds to the ECD domain of TrkB (Figure 3). Interestingly, deoxygedunin and alpha-dihydrogedunol (epoxy ring down) provoked GST-TrkB to bind HA-TrkB in cotransfected HEK293 cells, leading to TrkB receptor association and autophosphorylation. However, competition assay shows that deoxygedunin is unable to compete off BDNF bound to TrkB (data not shown). Presumably, it is due to its much weaker binding affinity (1.4 µM) than BDNF (K_d_ ∼10^−10^ M). Although the detailed molecular mechanism of how a ligand provokes Trk receptor activation remains unclear, a conformational change hypothesis has been proposed. For instance, a mutation at P203A can cause spontaneous dimerization and activation of TrkA [41], suggesting that ligand-provoked receptor dimerization might be through triggering conformational change. Conceivably, deoxygedunin binding to TrkB might elicit its conformational change, leading to its dimerization. Remarkably, although dihydrodeoxygedunol, 3-alpha-acetoxy-dihydro-deoxygedunin and 3-deoxo-3-beta-acetoxy-deoxydihydrogedunin failed to provoke HA-TrkB to bind GST-TrkB, the precipitated GST-TrkB was strongly phosphorylated by these chemicals. This partial agonistic effect fits with their weak stimulatory activity on TrkB receptor (Figure 1B, C and 2A, B). The maximal activation following ligand binding requires initial autophosphorylation of tyrosine residues 670, 674, and 675 on TrkB [42], [43]. Previous studies suggest the following model for Trk activation: in the ligand-unbound state, the activation loop blocks access of substrates to the active site of the kinase domain. Ligand binding permits autophosphorylation of the activation-loop tyrosines. Once phosphorylated, each of these forms specific charge-pair interactions with nearby standing positive charges. Such interactions in turn stabilize an “open” conformation in which the activation loop no longer blocks access of substrates to the kinase. This stabilized conformation now effectively phosphorylates inter- and intramolecular target [42]. Conceivably, the robust GST-TrkB phosphorylation might result from the intramolecular reaction in the stabilized conformation by the 3 chemicals mentioned above.

We have shown that both 7,8-DHF and deoxygedunin bind to the ECD of TrkB and provoke the receptor dimerization autophosphorylation. They both display comparable agonistic activity on TrkB receptor. Moreover, they both minic BDNF by exhibiting robust neuroprotective effect in stroke, neuroexcitotoxicity, and vestibular ganglia survival assays. In addition, these two compounds also display strong activity in learning and memory and depression in animal models. Forced swim test revealed that deoxygedunin might possess more robust antidepressant effect than 7,8-DHF (Fig. 6).

Taken together, these two small molecules basically share the same biological and therapeutic actions. Nonetheless, 7,8-DHF is water soluble, but deoxygedunin is not. The latter has more complicated structure scaffold than the former. The flavonoids are easier for medicinal modification to improve their biological effect than gedunin family members.

BDNF and TrkB receptor are targets for therapeutic intervention in various neurological diseases including neuroexcitotoxicity, stroke, depression, anxiety, neurodegeneration etc. Nevertheless, BDNF is not useful as a therapeutic agent because of its poor pharmacokinetic properties. To search for small molecules that possess robust TrkB agonistic activity, here, we invented a cell-based survival functional assay. Our high-throughput screen was focused on the neuronal survival function provided by the TrkB receptor. Only the compounds that selectively protect TrkB expressing cells but not parental cells without TrkB from apoptosis were subjected to next round functional analysis. Hence, the positive hits from the first round screen either directly activate TrkB as an agonist or facilitate the downstream survival machinery mediated by TrkB receptor. The second round screening in primary neurons and follow-up TrkB association and autophosphorylation analyses eliminate compounds that did not directly target TrkB receptor. Through the *in vitro* receptor/ligand binding assay, *in vivo* TrkB activation and neuronal survival experiments, we finally obtained a few potent and selective TrkB agonists that virtually mimic BDNF's biochemical and physiological actions, and 7,8-DHF and deoxygedunin are the most promising lead compounds. Employing a variety of animal models, we have established that deoxygedunin exhibits potent neuroprotective actions in kainic acid neuroexcitotoxicity and stroke animal models (see also Supplemental Materials S1 and Figure S3). Moreover, we found that vestibular ganglion loss in BDNF −/− pups was significantly blocked by the drug treatment (Figure 5). This finding demonstrates that TrkB agonists can protect vestibular ganglia from degeneration in BDNF-lacking mice. Further, we show that both 7,8-DHF and deoxygedunin display prominent antidepressant action in forced swim test, which is also TrkB dependent (Figure 6). BDNF/TrkB signaling is also associated with and required for the acquisition of classical conditioned fear in rodent models [34], [35], [36], [37], [38], [39]. Once again, we demonstrated that deoxygedunin enhances acquisition of conditioned fear, which is a BDNF-dependent learning process (Figure 7). Therefore, all of these animal models strongly support the notion that deoxygedunin mimics BDNF *in vitro* and *in vivo* and reveal remarkably therapeutic activities in various neurological diseases.

In CNS, physiological activities regulate local BDNF synthesis and secretion, providing neurotrophic support necessary for the BDNF-responsive tissues in a timely, dynamic fashion. In some regions, neurotrophins act by either a paracrine or an autocrine mechanism, while other neurons could compete for the same factor in the more classical long-range, target-derived paradigm [44], [45]. However, it is impossible for the small molecular TrkB agonists to meet the needs of TrkB activation in tissues that require temporal and spatial regulation by like BDNF or NT-4/5. The finding that 7,8-DHF and deoxygedunin rescue the survival of vestibular ganglia in BDNF −/− pups supports that these TrkB agonists partially supplement the deficiency of BDNF in mice. Undoubtedly, further chemical modification on these lead compounds to improve their agonistic activities and binding affinities might provide powerful tools for us to dissect the physiological functions of BDNF/TrkB signalings.

## Materials and Methods

### Ethics statement

TrkB^F616A^ mice, TrkB +/−, TrkA +/− and BDNF +/− C57BL/6 mice were bred in a pathogen-free environment in accordance with Emory Medical School guidelines. All animal experiments were performed according to the care of experimental animal guidelines from Emory University and were approved by Emory Institutional Animal Care and Use Committee (Protocol no. 060-2009).

### Cells and reagents

Human embryonic kidney HEK293 cell lines were grown in medium A (DMEM with 10% fetal bovine serum (FBS) and 100 units penicillin-streptomycin) at 37°C with 5% CO_2_ atmosphere in a humidified incubator [19]. Mouse septal neuron x neuroblastoma hybrids SN56 cells were created by fusing N18TG2 neuroblastoma cells with murine (strain C57BL/6) neurons from postnatal 21 days septa [19]. SN56 cells were maintained at 37°C with 5% CO_2_ atmosphere in DMEM medium containing 1 mM pyruvate and 10% FBS. T48 and T62 cells, stably transfected with rat TrkB in SN56 cells were cultured in the same medium containing 300 µg/ml G418 [19]. NGF and BDNF were from Roche. Phospho-Akt-473 or 308, Akt antibodies were from Cell Signaling. Anti-phospho-Erk1/2, anti-phospho-TrkA Y785 were from R & D Systems. Anti-TrkA antibody was from Santa Cruz. Anti-TrkB antibody was from Biovision. Anti-p-TrkB (816) was a gift from Dr. Moses Chao. Anti-p-TrkB 817 antibody was from Epitomics, and it was marked under each blot. The chemical library containing 2000 biologically active compounds was from The Spectrum Collection (MicroSource Discovery System, Inc. Gaylordsville, CT 06755). TrkB^F616A^ mice have been described previously [24]. [^3^H]Acetic acid, sodium salt was purchased from Perkin Elmer (specific activity: 75–150 mCi/mmol; concentration: 10 mCi/mL). Deoxygedunin was purchased from Gaia Chemicals, Inc. All other chemicals were purchased from Sigma or Alfa Aesar.

### Cell-based Screen

TrkB stable transfected SN56 cells (T48 cells) were seeded in a 96-well plate at 10,000 cells/well in 100 µl complete medium. Cells were incubated overnight, followed by 30 min pretreatment with 10 µM compounds in DMSO (10 mM stock concentration from The Spectrum Collection library). The cells were then treated with 1 µM staurosporine for 9 h. One h before the termination of the experiment, 10 µM MR(DEVD)2, a cell permeable caspase-3-activated fluorescent dye was introduced. Cells were fixed with 4% paraformaldehyde for 15 min. Cells were washed with PBS and incubated with 1 µg/ml of Hoechst 33342 for 10 min. Cover slides were washed with PBS, mounted, and examined using a fluorescence microscope.

### Preparation of 32-[^3^H]_3_deoxygedunin at Georgia Institute of Technology

[^3^H]Acetic acid, sodium salt (11 µmol, 0.17 mL, 0.17 mL of ethanol solution) is syringed into a heavy-walled glass vial bearing a magnetic stirrer. The ethanol is removed under vacuum and replaced with 0.5 mL of THF at 0°C. Isobutylchloroformate (3.0 µL, 23 µmol) was then added and the reaction mixture was stirred for 1 h at 0°C. A solution of 7-deacetyldeoxygedunin (5 mg, 11 µmol), prepared by acetyl deprotection of deoxygedunin with K_2_CO_3_ in MeOH, in 0.5 mL THF was then added dropwise. The reaction was stirrer for another 1 h. Solvent was removed under vacuum and the product was purified by preparative thin layer chromatography (SiO2; 1∶1 EtOAc:hexanes) to give 3 mg (58%) of 32-[^3^H]_3_deoxygedunin. Note: Preparation of 32-[^3^H]_3_deoxygedunin occurred only after preparation of deoxygedunin under identical reaction conditions in order to confirm product formation.

### TrkB dimerization Assay

HEK293 cells that were transfected with GST-TrkB and HA-TrkA or TrkB were washed once in PBS, and lysed in 1 ml lysis buffer (50 mM Tris, pH 7.4, 150 mM NaCl, 1 mM EDTA, 0.5% Triton X-100, 1.5 mM Na_3_VO_4_, 50 mM NaF, 10 mM sodium pyrophosphate, 10 mM sodium β-glycerophosphate, 1 mM phenylmethylsulfonyl flouride (PMSF), 5 mg/ml aprotinin, 1 mg/ml leupeptin, 1 mg/ml pepstatin A), and centrifuged for 10 min at 14,000× g at 4°C. The supernatant was transferred to a fresh tube. Transfected TrkB receptor was pulled down with glutathione beads, and the coprecipitated proteins were resolved on SDS-PAGE. The samples were transferred to a nitrocellular membrane, and immunoblotting analysis was performed with a variety of antibodies.

### Primary rat cortical or hippocampal neuron culture

Primary rat cortical or hippocampal neurons were prepared as follows. E17 rat pups were decapitated and cortex or hippocampus was extirpated, cross chopped and suspended by pipetting for separation in 5% fetal calf serum (FCS), 5% horse serum (HS) DMEM gently. The cell suspension was then centrifuged at 250× g for 5 min. This operation was repeated again. Cells were seeded into polyethyleneimine-coated 10 dishes and 12-well plates including coated-coverslips and incubated at 37°C in 5% CO_2_/95% air. After 3 h, the culture medium was changed to Neurobasal containing B-27 supplement (Invitrogen) and incubated for 4 days. For maintenance, a half medium is changed to fresh Neurobasal/B27 every 4 days. After 2 weeks, the cultured neurons are used in various experiments.

### Binding constant determination

Purified TrkB ECD or ICD proteins (10 µg/each) were incubated with different [^3^H-deoxygedunin] in 1 ml binding buffer (0.05 M Na/K phosphate buffer (pH 7.1), 200 mM NaCl) (1 nM [^3^H]deoxygedunin ∼82300 cpm) at 4°C for 10 min. After the incubation, the reaction mixture was loaded on filter paper. The mixture was washed with 3×5 ml Tris buffer (100 mM Tris, pH 7.1). The dried filter paper was put into a small vial and subjected to liquid scintillation counter analysis. The value of the dissociate constant and the number of sites were obtained from Scatchard plots by using the equation r/[L]free  =  n/Kd - r/Kd, where r is the ratio of the concentration of bound ligand to the total protein concentration and n is the number of binding sites.

### Deoxygedunin suppresses KA-induced neuronal cell death

Male C57BL/6 mice aged of 60 days were injected intraperitoneally (i.p.) with a single dose of either 20% DMSO in saline or KA (20 mg/kg) (Sigma, MO) or deoxygedunin (5 mg/kg) followed by KA. Animals were continually monitored for 2 h for the onset of seizure activity. For most of the mice, the seizure stopped a few hours after KA administration. At 5 days following treatment, animals were anesthetized and perfused with 4% paraformaldehyde in 0.1 M phosphate buffered saline. Brains were removed, post-fixed overnight and processed for paraffin embedding. Serial sections were cut at 5 m and mounted on slides (Superfrost-plus, Fisher). The slides were processed for TUNEL staining in order to assess the degree of DNA fragmentation in hippocampal regions. For KA treatment on TrkB F616A knockin mice, the mice were pretreated with saline or 1NMPP1 (16.6 ng/kg, i.p.) on day before the drug treatment. Deoxygedunin (5 mg/kg) was intraperitoneally administrated on mice. In 4 h, KA (20 mg/kg) was injected into the mice. Then the mice were supplied with drinking water containing 1NMPP1 (50 µM) for another 4 days. In day 5, the mice were sacrificed and the hippocampal regions were analyzed by immunoblotting.

### Cortex- Specific BDNF Deletion

The Cortex-Specific Cre mouse line was previously described as “transgenic line C” [46]. Briefly, coding sequence for Cre-recombinase (Cre-IRES-DsRed2) was placed downstream of a 3 kb cholecystokinin (CCK) promoter, linearized, purified, and microinjected into the pronuclei of one-cell C57/BL6 embryos, which were then implanted into pseudo-pregnant C57/BL6 females. The purification and injections were performed at the Emory University Transgenic Facility. Following verification of gene expression in the different transgenic lines [47], we crossed the cortex-specific “line C” to a floxed-stop lacZ reporter mouse line [48] as well as the floxed *BDNF* mouse line [49]. Region specific Cre gene expression and *BDNF* deletion were confirmed with *in situ* hybridization, x-gal staining for β-galactosidase expression, and western blot for BDNF protein levels.

### Immunofluorescent staining on primary neurons

Primary hippocampal neurons were seeded on poly-L lysine coated coverslips in 12-well dish. After 7 DIV, the neurons were treated with 100 ng/ml BDNF or variety of gedunin compounds (0.5 µM) for 30 min, and then washed with PBS. Cells were fixed with 3% formaldehyde in PBS at room temperature for 10 min. The cells were then permeabilized and blocked by 0.4% Triton X-100 and 2% FBS in PBS at room temperature for 15 min, washed with PBS three times and treated with anti-MAP2 (1∶200) and anti-phospho-TrkB (816) antibodies (1∶100). After staining with FITC- or Rhodamine-conjugated secondary antibody, the coverslips were mounted on slides. Fluorescent images were evaluated on an OLYMPUS IX71 fluorescence microscope.

### Immunohistochemistry staining

Brain tissues were fixed in 4% paraformaldehyde overnight followed by paraffin embedding. Sections of 6 µm were cut. For immunohistochemical staining, brain sections were deparaffinized in xylene and rehydrated in graded alcohols. Endogenous peroxidase activity was blocked by 3% hydrogen peroxide for 5 minutes and all slides were boiled in 10 mM sodium citrate buffer (pH 6.0) for 10 minutes. Phosphorylated Trk A, Trk A, phosphorylated Trk B, and Trk B were detected using specific antibodies and Zymed Histo-SP AEC kit (Invitrogen, USA). Slides were then counterstained with hematoxylin.

### Focal ischemia model

A total of 12 rats were used in the present study; 1 rat was excluded because of inadequate reperfusion. Focal cerebral ischemia was induced by occlusion of the right middle cerebral artery as previously described [50]. ***Drug Administration:*** The rats subjected to MCAO incurring ischemic insult <40% of baseline LDF were randomly assigned to receive either deoxygedunin (n = 4), 7,8-DHF (n = 4), or vehicle (n = 4) treatment. Deoxygedunin and 7,8-DHF were given at the dose of 5 mg/kg by i.p. injection 5 min prior to the onset of reperfusion. Rats in the vehicle group underwent the same experimental protocol, except that they received an identical volume/weight of vehicle only. Statistical analysis*:* All results were expressed as mean ± S,E,M. Mean ischemic lesion volume were analyzed using the Student's t-test. The criterion for statistical significance was set at p<0.05.

### Vestibular Ganglion dissection in BDNF −/− pups

The cochleae of various drugs-treated pups (P1 or P2 BDNF +/+ and −/− pups) were first fixed through cardioperfusion of 4% paraformaldehyde (in PBS). The cochlea was dissected out and postfixed in 1% osmium for 1 hr at room temperature. Samples were decalcified in 0.35 M EDTA (pH 7.5, in PBS) for 72 hrs at 4°C, followed by gradual dehydration in graded alcohols, infiltrated, and embedded in epoxy resin with the conventional protocols. Consecutive cochlear sections (5 µm in thickness) were cut with a microtome (Microm HM335E, GmbH) along the axis of the modiolus. Sections were stained with toluidine blue. Vestibular ganglions were identified by their location in the auditory internal meatus with the basal cochlear turn and the cochlear modiolus as morphological reference landmarks.

### Mouse conditioned fear studies

Following two-day habituation to testing context, wild-type C57Bl/6J mice (N = 28, male, 8–10 weeks old) were fear conditioned in eight identical startle response systems (SR-LAB, SDI) consisting of a nonrestrictive Plexiglas cylinder, 5.5 cm in diameter and 13 cm long, mounted on a Plexiglas platform which was located in a ventilated, sound-attenuated chamber. One hour prior to fear conditioning, mice received 8-OH-Deoxygedunin (N = 14, 5 mg/kg, i.p.) or vehicle (N = 14, 17%DMSO in PBS). Mice then received 5 tone – footshock pairings, with a 30 sec 12 kHz, 85dB tones which co-terminating with footshocks (intensity of 0.5 mA, 0.5 sec) with a 5 min intertrial interval, after which they were returned to their homecage. 24 and 48 hrs after training, mice were tested for freezing in rodent modular test chambers with an inside area of 30.5 cm ×24.1 cm ×21.0 cm as described previously [51], [52]. Three minutes after placing the mouse in the test chamber, 15 30 sec CS tones with an ITI of 1.5 min were delivered through a high-frequency speaker attached to the side of each chamber. Percentage time spent freezing during the CS presentations was calculated for each mouse using FreezeFrame (Coulbourn Instruments, #ACT-100).

## Supporting Information

Supplemental Materials S1 (0.04 MB DOC)

Figure S1 Chemical structures of gedunin derivatives. The first and last chemicals (beta-dihydrogedunol and alpha-dihydrogedunol) on the top row, the first one (dihydroxygedunol) in the middle row, and the third one on the bottom row (deoxygedunin) are the positive hits during cell-based screening and confirmed with primary cultures.
(0.66 MB TIF)

Figure S2 Deoxygedunin is orally active and it does not regulate Trk receptor expression in mouse brain. (A) Two-three months old mice (C57BL/6J) mice were orally injected with various doses of 7,8-DHF or deoxygedunin. The mice were sacrificed 2 h or 4 h after drug administration. The brain lysates were prepared and analyzed by immunoblotting. TrkB in mouse brain was orally activated by these two compounds with dosage as low as 1–5 mg/kg. (B) RT-PCR analysis of TrkA and TrkB receptors in mouse brain after deoxygedunin treatment. (C) p-TrkB 817 antibody can recognize phosphorylated human and mouse TrkB receptor. Human TrkB was transfected in SH-SY5Y neuroblastoma cells. The cells were treated with DMSO, BDNF (50 ng/ml) and 500 nM deoxygedunin for 15 min. TrkB was pulled down with pan-Trk antibody (Santa Cruz) and analyzed by immunoblotting with anti-p-TrkB 817. Mouse Trk receptor was immunoprecipitated with anti-pan-Trk from the lysates of mouse brains. The mice were i.p. injected with vehicle or 5 mg/kg deoxygedunin, and sacrificed 8 h after treatment.
(0.85 MB TIF)

Figure S3 Deoxygedunin displays potent therapeutic effects in cell death and stroke models. (A) Deoxygedunin decreases KA-induced apoptosis in mouse brain. The brain slides were analyzed with TUNEL assay. Green stands for apoptotic nuclei, which were also stained with DAPI; Kainic acid evidently initiated strong apoptosis in hippocampal CA3 region, which was substantially blocked by deoxygedunin (left panel). Quantitative analysis of apoptosis in the hippocampus (right panel). (B) Deoxygedunin is neuroprotective against stroke. TTC-stained coronal section from representative animals given either vehicle (60% DMSO) or deoxygedunin was shown. Infarcts are shown as pale (unstained) regions involving striatum and overlying cortex (left panel). Infarct volumes after 24 h MCAO is decreased after deoxygedunin treatment. The data are represented as mean ± SD; * (p<0.05)  =  significant difference compared to MCAO + Vehicle (right panel).
(2.21 MB TIF)

Figure S4 Deoxygedunin is mainly concentrated in rat brain olfactory bulb and hippocampus. Two-three months old mice (C57BL/6J) mice were intraperitoneally injected with 30 µl of [3H]-deoxygedunin (2×106 cpm)/DMSO/PBS solution. In 4 h, various brain regions (A) and different organs (B) and were analyzed by liquid scintillation counter.
(0.62 MB TIF)
