# Supplementary material for: Deoxygedunin, a Natural Product with Potent Neurotrophic Activity in Mice
Source: PLoS One. 2010 Jul 13;5(7):e11528. doi: 10.1371/journal.pone.0011528 (PMC2903477; doi:10.1371/journal.pone.0011528)
Supplement: Supplemental Materials S1 — (0.04 MB DOC) [file pone.0011528.s001.doc]

## Supplemental materials

## Deoxygedunin displays potent therapeutic effects on various neurological disorders

Kainic acid (KA) is a specific agonist for the kainate receptor. KA induces neuronal cell death in caspase-dependent and independent manners ^25-27^. To explore whether deoxygedunin can block the neurotoxicity initiated by KA, we intraperitoneally injected 5 mg/kg deoxygedunin into C57BL/6 mice, followed by 20 mg/kg KA. In 5 days, the mice were perfused and the brains were cut to a thickness of 5 µm and mounted on slides. TUNEL staining revealed that KA provoked enormous apoptosis in the hippocampus, which was substantially diminished by deoxygedunin (Supplemental Figure 3A, left panel). Quantitative analysis of apoptosis in the hippocampus revealed that deoxygedunin strongly decreased KA-induced apoptosis in hippocampus by 60% (Supplemental Figure 3A, right panel). To further determine the neuroprotective potential *in vivo*, deoxygedunin was tested in a transient middle cerebral artery occlusion (MCAO) stroke model in adult male rats. After 2 h MCAO followed by reperfusion, the animals received vehicle or deoxygedunin (5 mg/kg) 5 min prior to the onset of reperfusion. All animals included in the study survived the ischemic insult and treatment with deoxygedunin. A representative brain slice stained with TTC 24 h after MCAO in vehicle-treated and deoxygedunin-treated rats is shown (Supplemental Figure 3B, left panel). Area and volume measurements from TTC sections indicated that treatments with deoxygedunin significantly reduced infarct volumes in this transient ischemic model of stroke (Supplemental Figure 3B, right panel). These results concur with a previous report that pretreatment with intraventricular BDNF reduces infarct size after focal cerebral ischemia in rats and support the hypothesis of a neuroprotective role for BDNF in stoke ^7,28^. Taken together, deoxygedunin is a potent TrkB agonist, which prevents neuronal cell death and protects the neurodegeneration elicited by excitatory neurotoxicity and stroke.
