# Supplementary figures and images for: Deoxygedunin, a Natural Product with Potent Neurotrophic Activity in Mice
Source: PLoS One. 2010 Jul 13;5(7):e11528. doi: 10.1371/journal.pone.0011528 (PMC2903477; doi:10.1371/journal.pone.0011528)

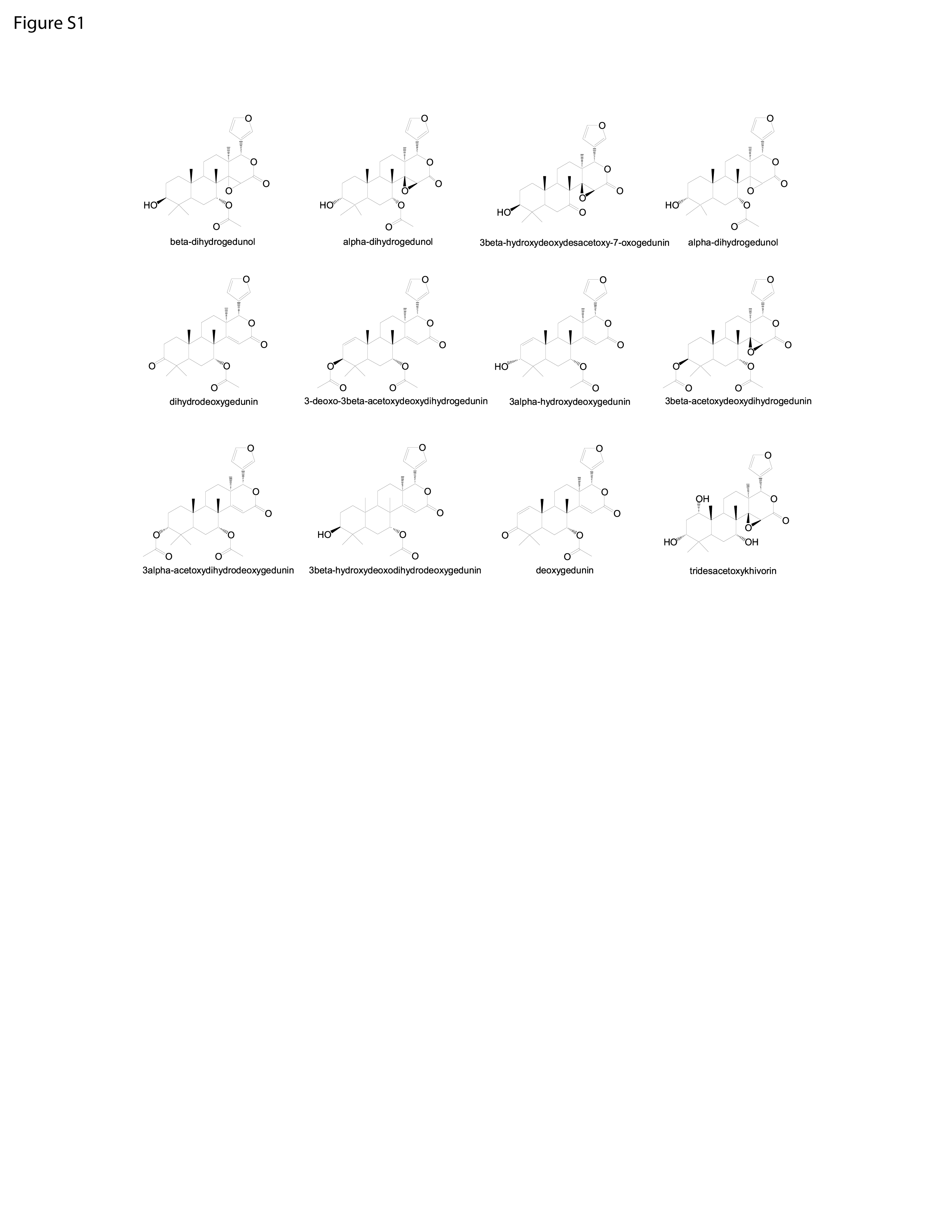

Supplement: Figure S1 — Chemical structures of gedunin derivatives. The first and last chemicals (beta-dihydrogedunol and alpha-dihydrogedunol) on the top row, the first one (dihydroxygedunol) in the middle row, and the third one on the bottom row (deoxygedunin) are the positive hits during cell-based screening and confirmed with primary cultures. (0.66 MB TIF) [file pone.0011528.s002.tif]

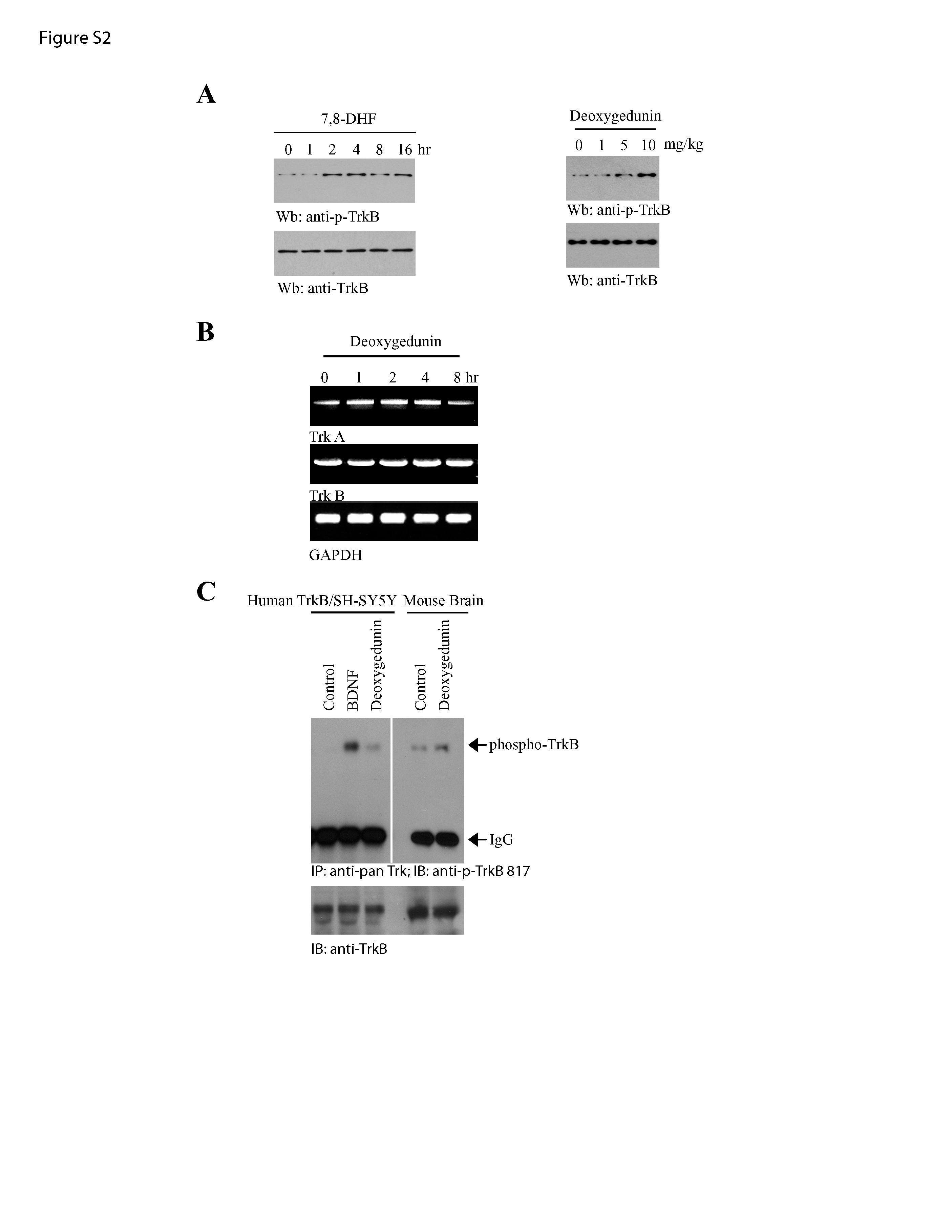

Supplement: Figure S2 — Deoxygedunin is orally active and it does not regulate Trk receptor expression in mouse brain. (A) Two-three months old mice (C57BL/6J) mice were orally injected with various doses of 7,8-DHF or deoxygedunin. The mice were sacrificed 2 h or 4 h after drug administration. The brain lysates were prepared and analyzed by immunoblotting. TrkB in mouse brain was orally activated by these two compounds with dosage as low as 1–5 mg/kg. (B) RT-PCR analysis of TrkA and TrkB receptors in mouse brain after deoxygedunin treatment. (C) p-TrkB 817 antibody can recognize phosphorylated human and mouse TrkB receptor. Human TrkB was transfected in SH-SY5Y neuroblastoma cells. The cells were treated with DMSO, BDNF (50 ng/ml) and 500 nM deoxygedunin for 15 min. TrkB was pulled down with pan-Trk antibody (Santa Cruz) and analyzed by immunoblotting with anti-p-TrkB 817. Mouse Trk receptor was immunoprecipitated with anti-pan-Trk from the lysates of mouse brains. The mice were i.p. injected with vehicle or 5 mg/kg deoxygedunin, and sacrificed 8 h after treatment. (0.85 MB TIF) [file pone.0011528.s003.tif]

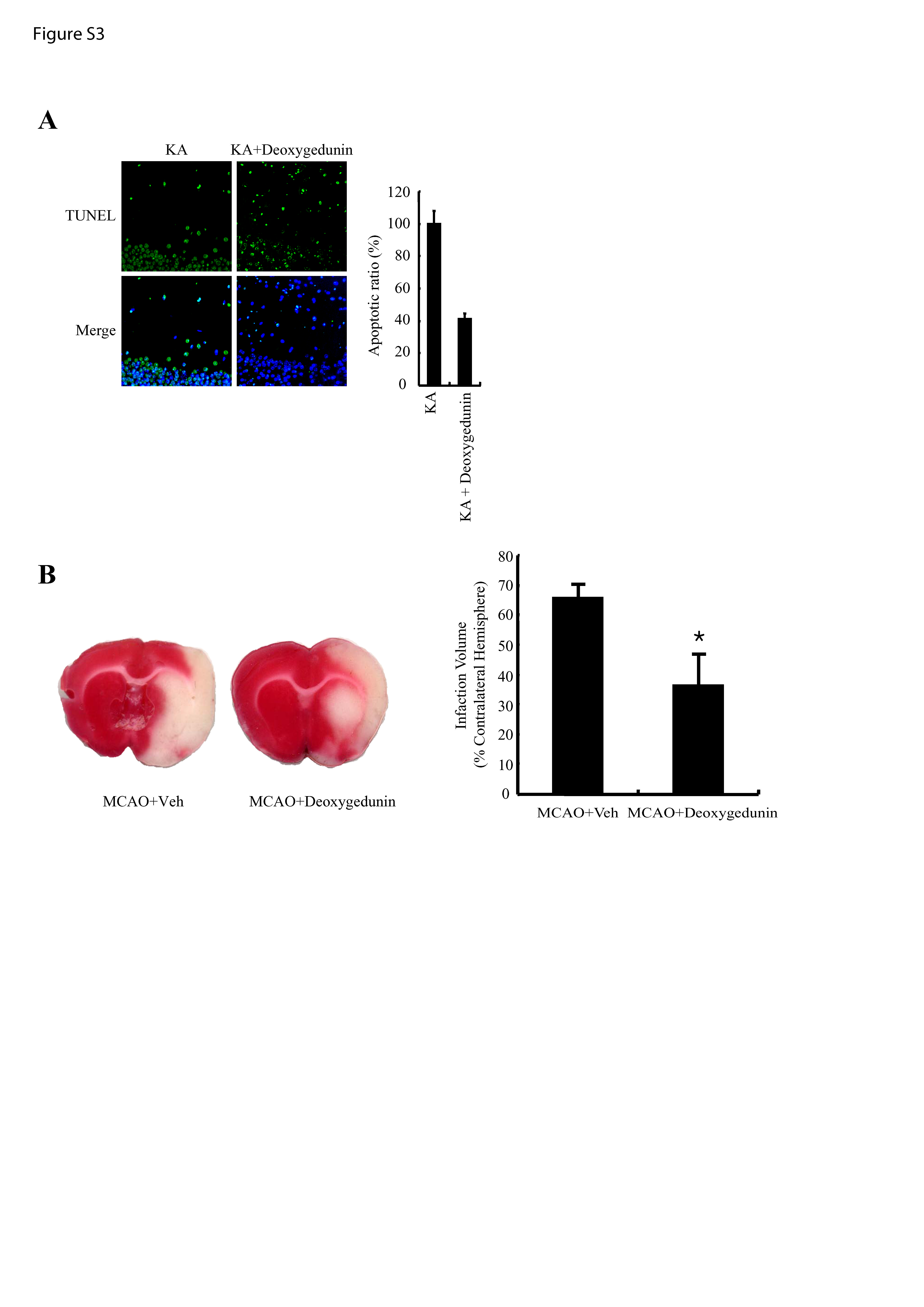

Supplement: Figure S3 — Deoxygedunin displays potent therapeutic effects in cell death and stroke models. (A) Deoxygedunin decreases KA-induced apoptosis in mouse brain. The brain slides were analyzed with TUNEL assay. Green stands for apoptotic nuclei, which were also stained with DAPI; Kainic acid evidently initiated strong apoptosis in hippocampal CA3 region, which was substantially blocked by deoxygedunin (left panel). Quantitative analysis of apoptosis in the hippocampus (right panel). (B) Deoxygedunin is neuroprotective against stroke. TTC-stained coronal section from representative animals given either vehicle (60% DMSO) or deoxygedunin was shown. Infarcts are shown as pale (unstained) regions involving striatum and overlying cortex (left panel). Infarct volumes after 24 h MCAO is decreased after deoxygedunin treatment. The data are represented as mean ± SD; * (p<0.05) = significant difference compared to MCAO + Vehicle (right panel). (2.21 MB TIF) [file pone.0011528.s004.tif]

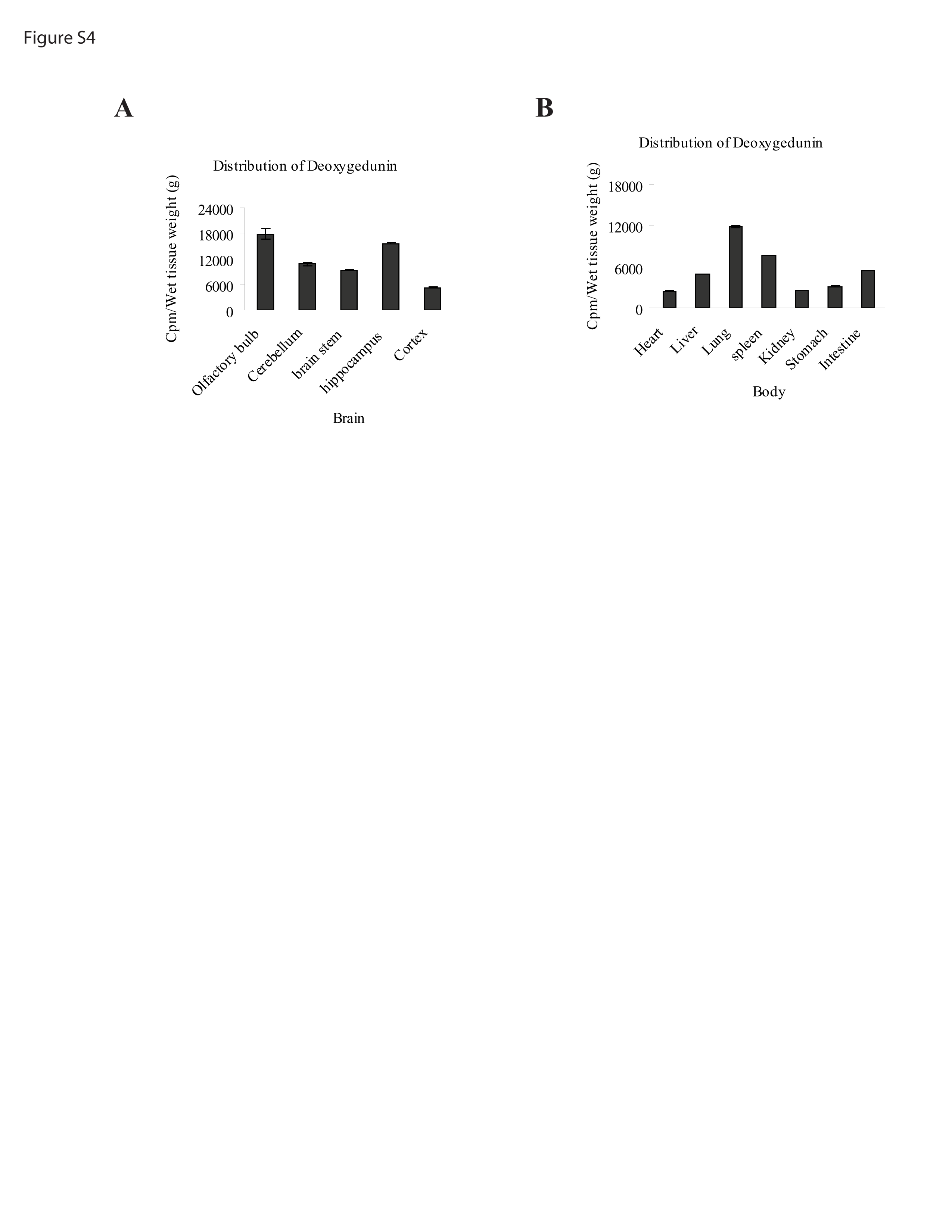

Supplement: Figure S4 — Deoxygedunin is mainly concentrated in rat brain olfactory bulb and hippocampus. Two-three months old mice (C57BL/6J) mice were intraperitoneally injected with 30 µl of [3H]-deoxygedunin (2×10^6^ cpm)/DMSO/PBS solution. In 4 h, various brain regions (A) and different organs (B) and were analyzed by liquid scintillation counter. (0.62 MB TIF) [file pone.0011528.s005.tif]
